# Supplementary material for: The Effect of N-Acetylation on the Anti-Inflammatory Activity of Chitooligosaccharides and Its Potential for Relieving Endotoxemia
Source: Int J Mol Sci. 2022 Jul 26;23(15):8205. doi: 10.3390/ijms23158205 (PMC9330575; doi:10.3390/ijms23158205)
Supplement: Supplementary file 1 [file ijms-23-08205-s001.zip › ijms-1808536-supplementary.pdf]

Supplementary material:

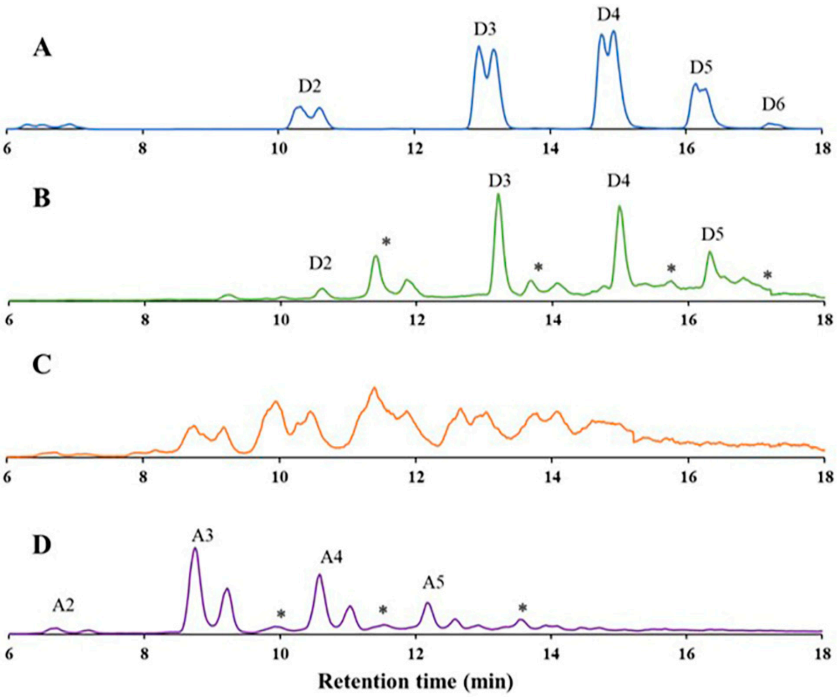

Figure S1. The HPLC spectra of four COS samples. A-D corresponding to COSs with DA of 0%, 12%, 50%, and 85%, respectively. Since the column we used is a hydrophilic column, the higher the number of amino groups, Samples with more amino groups appear later. In figure A and B, the retention time of (GlcN)2~(GlcN)6 are 10.5, 13.1, 14.8, 16.1 and 17.2 min, respectively. In figure D, the retention time of (GlcNAc)3~(GlcNAc)5 are 8.8, 10.6, and 12.2 min, respectively. Peaks labeled with * are supposed to be those N-acetylated chitooligosaccharides.

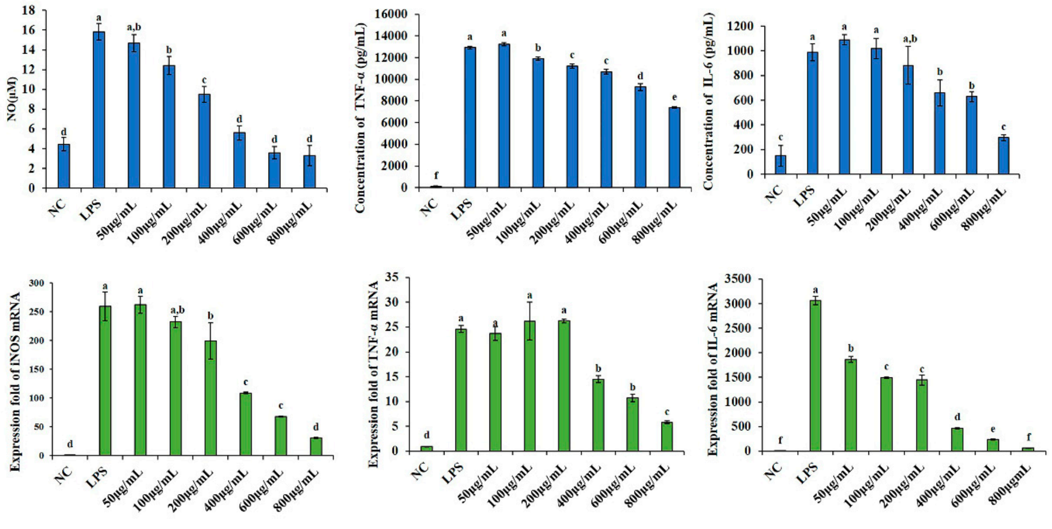

Figure S2. COS inhibited LPS-induced secretion of NO, IL-6 and TNF-α, as well as mRNA expression

levels of iNOS, IL-6 and TNF-α. No significant difference between the same letters. The cells treated with COS can effectively reduce the adverse effects of LPS in a concentration-dependent manner. When the concentration reaches 600-800μg/ml, it can even directly eliminate the effect of LPS.

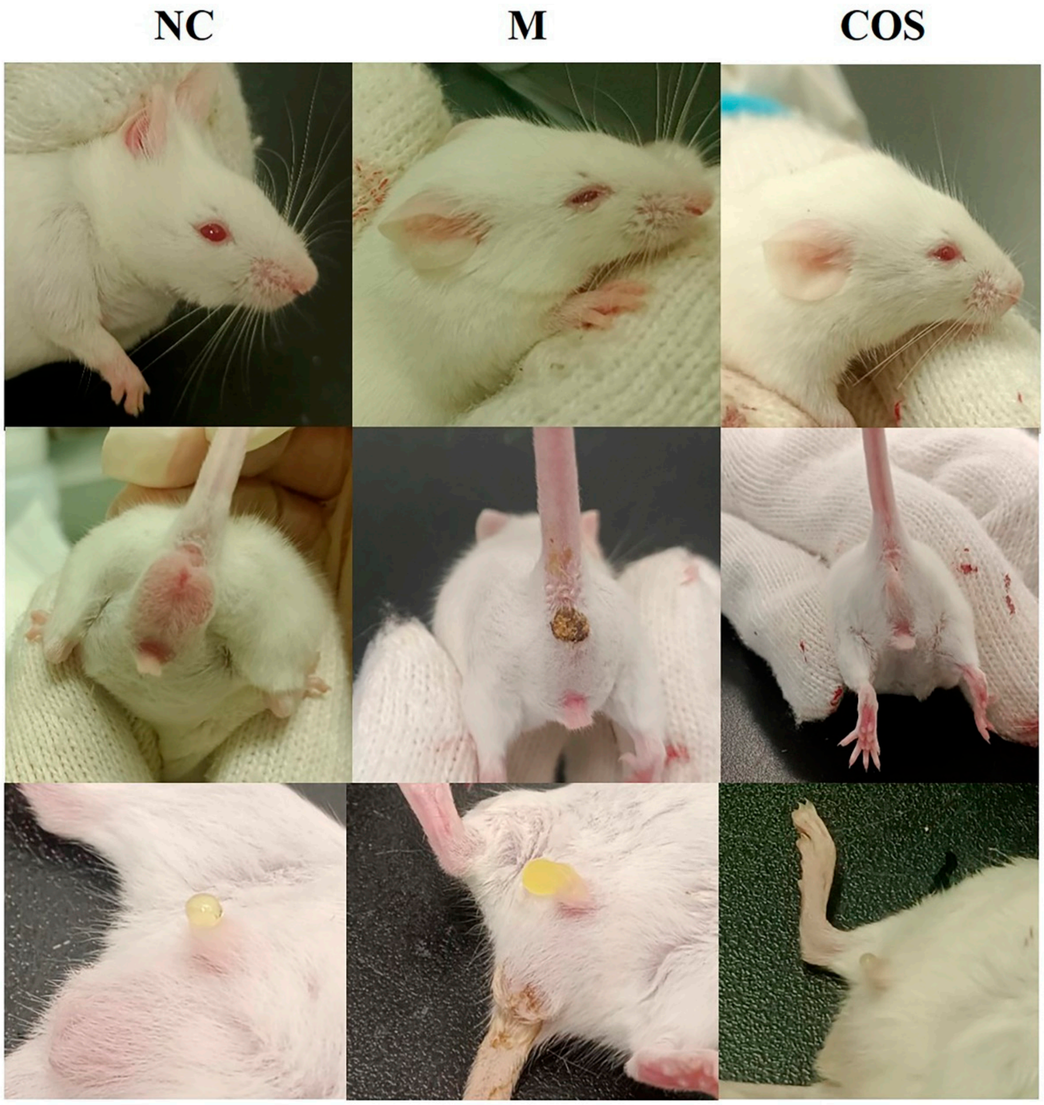

Figure S3. Behavior and characteristics of mice in different groups. NC represents the blank group; M represents the model group which challenged with LPS; COS group represents mice pretreated with 12% COS and then challenged with LPS. After the injection of LPS, the behavior and appearance characteristics of the mice in the model group showed obvious changes, such as sluggish movement, increased ocular exudate, and loose eyes. At the same time, the urine is turbid, the stool is not shaped, and the color of the stool is black. However, mice pretreated with COS at a concentration of 100 mg/kg significantly improved these above phenomena.
